# Synthesising practice guidelines for the development of community-based exercise programmes after stroke

**DOI:** 10.1186/1748-5908-8-115

**Published:** 2013-10-01

**Authors:** Leon Poltawski, Charles Abraham, Anne Forster, Victoria A Goodwin, Cherry Kilbride, Rod S Taylor, Sarah Dean

**Affiliations:** 1 University of Exeter Medical School, Veysey Building, Salmon Pool Lane, Exeter EX2 4SG, UK; 2 University of Exeter Medical School, St Luke’s Campus, Magdalen Rd, Exeter, UK; 3 Academic Unit of Elderly Care and Rehabilitation (University of Leeds), Bradford Institute for Health Research, Bradford Royal Infirmary, Duckworth Lane, Bradford, UK; 4 Centre for Research in Rehabilitation, Brunel University London, Mary Seacole Building, Kingston Lane, UB8 3PH, Middlesex, UK

**Keywords:** Guidelines, Synthesis, Stroke, Rehabilitation, Exercise

## Abstract

**Background:**

Multiple guidelines are often available to inform practice in complex interventions. Guidance implementation may be facilitated if it is tailored to particular clinical issues and contexts. It should also aim to specify all elements of interventions that may mediate and modify effectiveness, including both their content and delivery. We conducted a focused synthesis of recommendations from stroke practice guidelines to produce a structured and comprehensive account to facilitate the development of community-based exercise programmes after stroke.

**Methods:**

Published stroke clinical practice guidelines were searched for recommendations relevant to the content and delivery of community-based exercise interventions after stroke. These were synthesised using a framework based on target intervention outcomes, personal and programme proximal objectives, and recommended strategies.

**Results:**

Nineteen guidelines were included in the synthesis (STRIDES; **ST**roke **R**ehabilitation **I**ntervention-**D**evelopment **E**vidence **S**ynthesis). Eight target outcomes, 14 proximal objectives, and 94 recommended strategies were identified. The synthesis was structured to present best practice recommendations in a format that could be used by intervention programme developers. It addresses both programme content and context, including personal factors, service standards and delivery issues. Some recommendations relating to content, and many relating to delivery and other contextual issues, were based on low level evidence or expert opinion. Where opinion varied, the synthesis indicates the range of best practice options suggested in guidelines.

**Conclusions:**

The synthesis may assist implementation of best practice by providing a structured intervention description that focuses on a particular clinical application, addresses practical issues involved in programme development and provision, and illustrates the range of best-practice options available to users where robust evidence is lacking. The synthesis approach could be applied to other areas of stroke rehabilitation or to other complex interventions.

## Background

The evidence base for rehabilitation after stroke is expanding rapidly [1]. Drawing on this evidence, numerous guidelines have been developed by expert bodies, to facilitate development of best clinical practice [2,3]. Many guidelines are comprehensive in coverage, addressing all phases in recovery from stroke and the many disciplines involved in providing post-stroke care [2-4]. However, the existence of practice guidelines does not in itself guarantee their implementation. Even high quality guidelines, developed according to rigorous standards and presenting unambiguous recommendations based upon robust evidence, may fail to influence practice for a variety of reasons. These may include factors intrinsic to the guidelines themselves, such as their user-friendliness and relevance to the user organisation [5-7]. They may also relate to the environment in which they are applied, for example organisational structures, service resource limitations and professional awareness [6,8-10].

A number of strategies have been suggested to reduce barriers to guideline implementation. One is to provide guidelines in multiple formats targeting different professional groups; some stroke practice guidelines do this by providing separate listings of recommendations for nursing and allied health professionals [4]. Another strategy is to adapt guidelines according to context such as a particular healthcare setting or application [9,10]. This involves identifying a clinical application or question, extracting relevant recommendations from existing guidelines, and re-presenting them in a format that is appropriate to the context [11]. Adapting existing guidelines enables consideration of a variety of recommendations produced in different cultural and organisational contexts, and avoids unnecessary duplication of effort [11]. The ADAPTE initiative has developed formal and detailed methods for this process [12]. However, it provides limited guidance on the process of 'customisation’ - integrating recommendations from guidelines with varying concerns and terminologies and which may not explicitly address the application of interest. This is particularly pertinent when guidelines are being adapted to inform the development of complex interventions that involve multiple components and delivery issues. In such cases, adapting existing guidelines involves the synthesis of recommendations into an overarching structure selected to facilitate programme development. We use the term 'synthesis’ because the process goes beyond the extraction and re-ordering of practice recommendations; it requires the development of a thematic framework into which the recommendations are placed, and may involve an element of conceptual translation.

The synthesis of practice guidelines for specific clinical applications is relatively uncommon. A recent example focused on the assessment and management of low back pain [13]. Using a systematic review methodology, data were extracted from ten guidelines and synthesised to produce tables of recommended diagnostic and treatment options, arranged under headings of primary and secondary care. None of the individual guidelines contained all of these recommendations, which supports the case for synthesising multiple publications. The authors note the lack of guidance on the quantity or 'dose’ of therapy, and suggest that guidelines often ignore practical realities faced by clinicians [13]. The synthesis did not address issues such as the clinical setting, who provides the interventions, and what level of expertise is required [9]. In rehabilitation, these and other contextual factors such as personal beliefs, differences in goals, and power relationships between therapists and client can significantly influence outcomes [14-16]. Theoretical models have been developed indentifying contextual factors as both moderators and mediators of rehabilitation outcomes [17,18], and guidelines for the development and evaluation of complex interventions suggest that these factors should be identified and described in accounts of interventions [19,20]. These principles are also supported by realist approaches to evaluation and evidence synthesis for complex interventions, in which effectiveness is seen as highly contingent on context: how, to whom, and in what circumstances interventions are implemented [21,22].

Thus, there is a need for application-specific guidelines that address both the content of interventions and the context in which they are delivered. A synthesis of guidelines created for this purpose [7,16-22] may be more credible and useful to potential users since it is tailored to a particular need and takes account of the many factors that may influence outcome [12]. Synthesising guidelines for the purpose of programme development is not a well-developed methodology, and there is a need to gain and reflect on experiences of doing so. We conducted a synthesis as part of development work for a clinical trial of a community-based exercise programme for stroke survivors. Such programmes are increasingly being offered to facilitate regular engagement in exercise by long term stroke survivors and so to improve their health outcomes [23-26], but the evidence for particular types of multi-component programme is presently limited. To help develop an intervention that is congruent with current best evidence, we synthesised relevant guideline recommendations regarding programme content, delivery and other contextual factors. The aim was to develop a synthesis that places recommendations in a conceptually coherent structure that can inform programme development. The purpose of this paper is to describe the process and outcome of the synthesis, and to discuss the potential value of this approach to others who wish to develop comprehensive syntheses of guidelines to assist the development and evaluation of other forms of complex intervention.

## Methods

**Figure 1:**
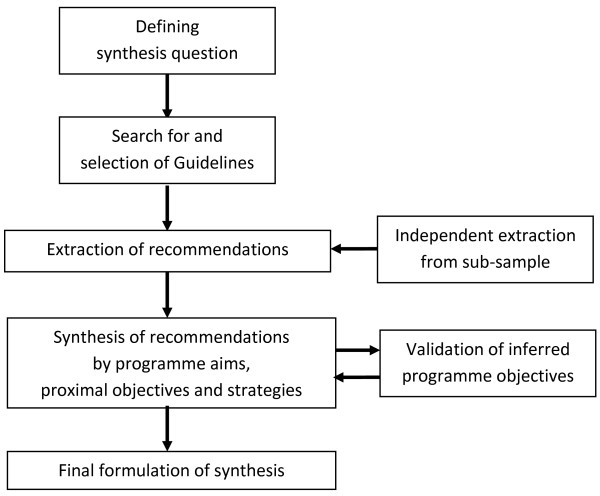
Synthesis process.

The synthesis used some of the elements described in the ADAPTE approach: defining the health question, searching for and screening guidelines, selecting recommendations, and creating a customised guideline [12] (see Figure 1). Data extraction and the synthesis process were informed by an approach called Intervention Mapping [27], which has been used by others to structure the development of complex health interventions [27-31], and is described later in this section.

### Defining the health question

The question we addressed in this synthesis was: how should community-based exercise programmes for long term stroke survivors be structured, in terms of content and delivery, to maximise their effectiveness?

### Search for guidelines

We searched for stroke practice guidelines (sets of recommendations generated by expert panels, regarding the content and delivery of interventions) published between 2000 and July 2012. We consulted the Medline and Cinahl bibliographic databases, the National Guideline Clearinghouse (http://www.guideline.gov), and Google Scholar. The search strategy used combinations and variations of the terms 'practice guideline,’ 'stroke,’ 'rehabilitation’ and 'exercise,’ and is described in Additional file 1. Free text searches using Google were also employed.

### Eligibility criteria

Guidelines were included if they made recommendations regarding either the use of physical exercise by adults after stroke, or the delivery of such interventions through community-based programmes. Exercise was defined as the use of structured, repetitive physical activities to maintain or enhance physical functioning [32]. Only guidelines developed or endorsed by national or professional organisations or bodies were included, and those concerned solely with acute care or primary prevention were excluded. Due to resource limitations, we excluded publications that were not available in English or which had to be purchased. Where guidelines had been superseded by, or incorporated into, a more recent publication from the same organisation, the latest publication was used. One reviewer (LP) conducted the search, and two (LP and SD) discussed and agreed which guidelines should be included in the synthesis. The ADAPTE process advocates quality assessment of guidelines, and suggests that quality scores may be used as eligibility criteria [12]. However, we decided that, for the purposes of this synthesis, the strength of evidence for recommendations was the key factor in informing practice, and so focussed on this rather than the methodological quality of the guidelines themselves.

### Data extraction

Both data extraction and analysis drew on an approach developed to assist in the planning of complex health interventions, called Intervention Mapping [27]. This approach proposes that interventions are characterised in terms of target outcomes, proximal objectives, methods and practical strategies. Target outcomes are the long term benefits that the intervention is designed to achieve for its participants; proximal objectives are the changes in the individual participant and their environment that are necessary to obtain the outcomes; and practical strategies are the means (or delivery modes) that may be employed to serve these objectives [27]. In Intervention Mapping, methods and strategies follow from a theoretical understanding of underlying regulatory processes. The approach has been used to aid the development of a range of complex health interventions, and it specifically seeks to incorporate service delivery and other contextual factors into intervention descriptions [30,31]. We used its principles to guide data extraction by selecting statements or recommendations relating to the aims, objectives, content and delivery of exercise-based interventions. Although the approach also involves the identification of theoretical underpinnings for strategies [27], we did not extract statements relating to theory in guidelines because we were primarily concerned with practice recommendations.

Our focus was on exercise programmes that can be delivered in community settings without the requirement for specialist clinical equipment. Therefore, we did not extract recommendations relating to the use of robots, partial-bodyweight support therapy, mirror boxes etc., but did include those requiring equipment that would be available in a typical community-based gym. Where guidelines cited and/or graded evidence in support of their recommendations, the type and grading of the evidence was recorded. We included both research-evidenced recommendations and those based on expert consensus alone because, in the absence of robust evidence, programme planners and practitioners may still be guided by the experience and views of experts. One reviewer (LP) extracted data from all guidelines, and a second reviewer (SD) verified the process by independent data extraction from four of the guidelines. Any differences were discussed by the reviewers, and additional material was included if agreed to be relevant.

### Synthesis of recommendations

Data were analysed thematically, using a form of template analysis in which data are initially. Categorised using predefined themes and then organised iteratively under a hierarchy of emerging sub-themes [33]. In this case, the predefined themes used Intervention Mapping terms: target outcomes, proximal objectives and strategies. Most of the guidelines were not specifically concerned with community-based exercise programmes, and few made explicit statements regarding the proximal objectives of such programmes. Thus, most data were initially classified under the 'strategies’ theme. Using an iterative approach, these strategies were grouped and re-grouped into a series of sub-themes, which were then expressed as proximal objectives.

One reviewer (LP) conducted the first iteration of this process using all the extracted data; subsequent iterations were developed through discussion with three other reviewers (SD, VG, CK) with rehabilitation expertise and who were familiar with a sample of the included guidelines, and one reviewer (CA) with expertise in Intervention Mapping methodology. To validate the inference of proximal objectives from the recomendations, two reviewers (LP and SD) independently inspected four guidelines to judge whether the proximal objectives were explicitly or implicitly present in them.

The guidelines used a variety of systems to grade the evidence cited to support their recommendations. For the synthesis, the gradings were reclassified under a single simple system that had been employed by one of the guidelines [34]: multiple randomised control trials (RCTs) and meta-analyses are given the highest grading (1), followed by single RCTs and non-randomised studies (2), and finally expert consensus, case studies, and standards of care (3).

## Results

**Figure 2:**
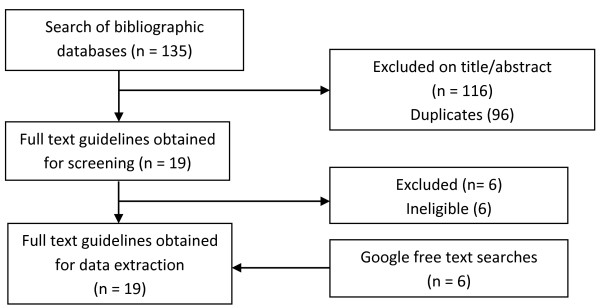
Search and screening flow chart.

The search of bibliographic databases identified 135 potentially relevant publications, which were screened initially by title and abstract, then by full text (see Figure 2). A total of 13 guidelines meeting the eligibility criteria were included, along with 6 further guidelines obtained through Google free text searches [1-3,34-49]. They were from Australia, Canada, a European network, the Netherlands, New Zealand, Singapore, South Africa, the United Kingdom and the United States. Three of these were produced by the American Heart Association but targeted different audiences with distinct recommendations. The guidelines varied considerably in scope, some providing only general endorsement of physical exercise (*e*.*g*., [36,44]), others making more specific and detailed recommendations [2,3]. The median number of relevant recommendations abstracted per guideline was 15, although 2 guidelines made only one recommendation each relevant to the synthesis topic [46,49].

The guidelines used a variety of formats, *e*.*g*., being structured according to the phases of stroke recovery, the concerns of each health professional group, or the types of intervention that could be provided. Terminologies also differed, for example in the naming of forms of exercise or types of health practitioner. Most addressed the continuum of care from acute to chronic phases, but two focused specifically on community-based interventions for longer-term stroke survivors [35,42]. These provided the majority of delivery-related recommendations, but did not mention several content and delivery strategies suggested in other guidelines, such as the use of mental rehearsal and tele-training. Eight target outcomes of exercise-based interventions were explicitly identified in one or more guidelines (see Table 1). Three of these related to body structure and function, and five to activities and participation in life roles. A total of 94 recommendations were identified, and 14 proximal objectives were either explicitly stated in at least one guideline or formulated from themes emerging from the data synthesis.

**Table 1:** Target programme outcomes

	
Reduced risk of stroke [2,34,36,37,39,40,45,47],[48]	Optimisation of functional ability [2,3,34,37,40,45]
Increased cardiovascular fitness [2,35,37,39,40,44]	Social participation to the extent desired [45,48]
Enhanced mobility [2,36,37]	Life-long involvement in regular physical exercise [2,35,41,42,45,47,48]
Reduced risk of falls [34,36,37,44]	Self-management of physical exercise [48]

Tables 2 and 3 summarise the proximal objectives and strategies for programme content and delivery respectively. Recommendations relating to contextual factors appear in both tables: environmental factors (such as family support) are addressed in Table 2, while personal factors (such as individual attitudes) are addressed by several strategies linked to objectives 6, 7 and 8. The tables also indicate the highest level of evidence cited in any guideline for each strategy. The strength of supporting evidence varied considerably: many of the recommendations for content were based on systematic reviews, whereas most of those relating to service delivery relied on qualitative studies or expert opinion. More detailed tables, providing page and section references for each recommendation, and any evidence gradings provided in each guideline, are available in Additional files 2 and 3 respectively.

**Table 2:** Proximal objectives and strategies for programme content

**Proximal objectives & recommended strategies**		**Ev**	**count**	**Au**	**Ca1**	**Ca2**	**Ca3**	**Ca4**	**Ca5**	**Eu**	**Ne**	**NZ**	**SA**	**Si**	**UK1**	**UK2**	**UK3**	**US1**	**US2**	**US3**	**US4**	**US5**
				[41]	[37]	[42]	[38]	[43]	[1]	[44]	[3]	[48]	[36]	[49]	[45]	[47]	[35]	[39]	[40]	[34]	[2]	[46]
***1***	***Increase muscle strength***		10	X		X		X			X	X			X		X	X	X		X	
	a. Lower limb strengthening	1	8			X		X	X		X						X		X	X	X	
	b. Upper limb strengthening	1	10		X	X		X	X		X				X		X		X	X	X	
	c. Trunk and core muscle strengthening	2	3			X											X		X			
***2***	***Increase aerobic endurance***		12		X	X		X	X	X	X	X			X		X	X	X		X	
	a. Treadmill training	1	6		X	X		X			X								X		X	
	b. Use of static bicycle	1	3			X											X		X			
	c. Other large muscle group aerobic activity	2	2			X											X					
***3***	***Regain and maintain normal joint range of movement***		5			X									X		X		X		X	
	a. Lower limb stretching	2	3		X	X											X					
	b. Upper limb stretching	1	4		X	X			X		X											
	c. Trunk stretching	2	2			X											X					
	d. Lower limb movement through range	2	2		X												X					
	e. Upper limb movement through range	2	3		X		X										X					
	f. Unspecified stretch/ range of movement exercises	3	5			X	X								X				X		X	
***4***	***Enhance sensorimotor functions required for functional activity***		7		X	X	X								X		X	X	X			
	a. Standing balance training	1	5			X		X	X								X				X	
	b. Sitting balance training	1	4			X		X			X										X	
	c. Aquatic balance training	1	2						X												X	
	d. Unspecified balance training	1	3						X						X				X			
	e. Cyclic movements of paretic arm	2	3								X						X				X	
	f. Proprioceptive & kinaesthetic training	2	6		X		X	X			X	X									X	
	g. Mental rehearsal of upper limb movements	1	8	X	X		X	X	X			X			X						X	
***5***	***Enhance functional ability***		12	X	X	X	X	X			X	X			X		X		X	X	X	
	a. Standing up & sitting down practice	1	7		X	X		X			X	X			X		X					
	b. Step training / stair climbing	1	5			X		X									X		X		X	
	c. Walking (including treadmill training)	1	11	X	X	X		X	X			X			X		X		X	X	X	
	d. Walking with rhythmic cueing	1	6	X				X	X		X	X									X	
	e. Paretic upper limb task-related training	1	11	X	X		X	X	X		X	X			X		X			X	X	
	f. Bilateral upper limb task-related training	1	5	X				X				X			X						X	
***6***	***Establish & maintain motivation for regular physical exercise***		6	X		X						X			X	X	X					
	a. Address personal beliefs & attitudes	2	1														X					
	b. Promote personal goal-setting	2	3									X			X		X					
	c. Use motivational interviewing	2	1												X							
	d. Promote use of personal reflective diaries	3	1														X					
	e. Promote use of personal exercise record including repetitions, load and time spent	3	2														X	X				
	f. Use positive feedback	2	1												X							
	g. Emphasise enjoyment	3	2			X											X					
***7***	***Develop self-management skills for ongoing physical exercise***		1			X																
	a. Educate for self-monitoring for adverse events	3	1														X					
	b. Promote active problem-solving	3	3									X			X						X	
	c. Develop self-efficacy skills	3	2			X									X							
	d. Encourage self-monitoring to set appropriate exercise levels	3	2			X											X					
	e. Encourage independent practice of exercises	3	2			X											X					
	f. Provide guidance booklets	3	1														X					

Ev = highest level of evidence presented for strategy.

n = number of guidelines explicitly including objective or strategy.

Au = Australia; Ca = Canada; Eu = Europe; Ne = Netherlands, NZ = New Zealand; SA = South Africa; Si = Singapore.

**Table 3:** Proximal objectives and strategies for programme delivery

**Proximal objectives & recommended strategies**		**Ev**	**n**	**Au**	**Ca1**	**Ca2**	**Ca3**	**Ca4**	**Ca5**	**Eu**	**Ne**	**NZ**	**SA**	**Si**	**UK1**	**UK2**	**UK3**	**US1**	**US2**	**US3**	**US4**	**US5**
				[41]	[37]	[42]	[38]	[43]	[1]	[44]	[3]	[48]	[36]	[49]	[45]	[47]	[35]	[39]	[40]	[34]	[2]	[46]
***8***	***Personalise programme to individual***		5														X	X	X	X	X	
	a. Multidimensional pre-assessment conducted by healthcare professional addressing health status, cautions, contraindications and risks	2	7			X						X					X	X	X	X	X	
	b. Pre-programme assessment by trainers to enable individualisation of programme	3	3				X										X	X				
	c. Adapt programme content to personal situation and goals	2	8	X		X						X			X		X	X		X	X	
	d. Evaluate programme effects on individual, including satisfaction, functional gains, personal goals, resource use, energy levels	3	3														X	X			X	
	e. Supplement group classes with individual sessions	3	1														X					
	f. Sub-divide group classes according to disability levels	3	1														X					
	g. In group classes, conduct functional strengthening exercises together to allow individual monitoring	3	2														X	X				
	h. Intensity should be adjusted to the individual	3	4			X									X		X		X			
	i. Pre-programme ECG assessment for exercise level	3	1																X			
	j. If maximal heart-rate is unknown, use low intensity but increase training frequency/duration	3	3			X												X	X			
	k. Shorter, more frequent exercise for frail or deconditioned	3	1			X																
	l. Use of memory aids where necessary	3	3									X			X		X					
***9***	***Ensure dosage is sufficient to establish and maintain benefits***		2							X		X										
	a. At least 3 days/week physical exercise	1	1														X					
	b. 20-30 minutes daily moderate intensity physical exercise	3	2											X	X							
	c. Progression: increase load / required effort over time	1	5	X		X									X		X				X	
	d. Aerobic exercise 20–60 minutes, 3–7 days/week; continuous or accumulated	2	2		X														X			
	e. Cardiovascular endurance should be large proportion of activity	3	1																X			
	f. Strengthening exercises: 4–10 types, 2–3 days/week	3	3			X											X		X			
	g. Flexibility exercises: 2–3 days/week	3	2			X													X			
	h. Coordination & balance exercises: 2–3 days/week	3	2			X													X			
	i. Upper limb exercises 1 hour, 6 days/week	3	1		X																	
	j. Warm-up: 15–20 minutes including range of movement and large muscle group activity	2	2														X	X				
	k. Aerobic warm-up and cool-down, 3–5 minutes at lower intensity	2	2			X											X					
	l. Aerobic: up to 10 exercises alternating cardiovascular & local muscle endurance	3	2														X	X				
	m. Include home exercises to increase dose	3	4			X	X					X					X					
***10***	***Structure programme to facilitate ongoing regular physical exercise***		4	X		X											X				X	
	a. Pre-programme contact to discuss any programme barriers	3	3			X											X		X			
	b. Peer/volunteer to accompany to first one or two sessions	3	1														X					
	c. Minimal use of equipment to facilitate home practice	3	1														X					
	d. Promote family / carer involvement	1	10	X		X	X					X	X		X			X	X	X	X	
	e. Use peer mentoring	2	2									X					X					
	f. Use group format for social support	3	1														X					
	g. Provide opportunities to socialise before and after training	3	2														X	X				
	h. Use of mixed media including internet-based and tele-training	2	4									X						X			X	X
	i. Locate at home or centre according to personal circumstances / preferences	1	5	X			X		X									X			X	
	j. Locate in own residential environment	1	3					X			X		X									
	k. Provision of transport where necessary, or locate near good public transport links	3	5	X		X									X		X	X				
	l. Convenient time	3	1			X																
	m. Ongoing programme provision	3	2			X											X					
	n. Sign-post to other relevant services / facilities	3	4	X								X			X						X	
***11***	***Ensure adequate staffing numbers to provide safe and effective training***		2							X							X					
	a. Instructor: participant ratio: 1:3 to 1:5	3	1			X																
	b. Instructor: participant ratio: up to 1:8 depending on mix & time since started exercising	3	2														X	X				
	c. Supernumerary volunteers or trainees to take part in sessions	3	1														X					
***12***	***Ensure staff are adequately trained for client group***		1														X					
	a. Delivered by instructors with knowledge and training in exercise and stroke	3	3			X										X	X					
	b. Provide in-service training to instructors	3	1														X					
	c. Ensure stroke-awareness training of frontline staff in course venue	3	1														X					
***13***	***Integrate programme into stroke pathway***		3					X									X			X		
	a. Develop partnership agreements between stakeholders	3	1														X					
	b. Referral by healthcare practitioner using clear eligibility criteria	3	2			X											X					
	c. Encourage referring practitioner to visit programme	3	1														X					
	d. Established procedures for transferring responsibilities from referrers to trainers	3	2														X	X				
	e. Ongoing communication with (and feedback to) other stakeholders including healthcare professionals, service commissioners, local stroke networks	3	2														X	X				
	f. Referral for other treatments where appropriate	3	2														X	X				
***14***	***Ensure adequate programme governance***		1														X					
	a. Oversight by management group	3	1														X					
	b. Plan for programme evaluation	3	2			X											X					
	c. Use procedures for recording and reporting adverse events	3	2			X											X					
	d. Follow data protection procedures	3	1														X					
	e. Obtain and check ongoing consent	3	2			X											X					

Ev = highest level of evidence presented for strategy.

n = number of guidelines explicitly including objective or strategy.

Au = Australia; Ca = Canada; Eu = Europe; Ne = Netherlands, NZ = New Zealand; SA = South Africa; Si = Singapore.

### Programme content

Seven proximal objectives focusing on the individual participant, along with 38 associated strategies, were identified in the synthesis. The objectives related to both physical outcomes, such as strength, aerobic fitness and functional ability, and to psychological outcomes, such as motivation and exercise self-management skills. The most commonly recommended exercises – with the highest level evidence – focussed on upper and lower limb strengthening, cardiovascular fitness and task-related practice. Treadmill training was frequently recommended both for improving aerobic endurance and to enhance walking performance. Several guidelines recommended and cited high level evidence for balance training, but there was less agreement about other sensorimotor strategies, *e*.*g*., mental rehearsal of movements and tasks, which received strong support from some guidelines [43,45] but less from others [2,42]. Stretching and range of movement exercises were advocated by several, but had the lowest levels of supporting evidence.

Several guidelines recommended a combination of aerobic endurance, strength, functional practice, and balance exercises for long term stroke survivors [3,37,40,43], but one claimed that there was no strong evidence to recommend any particular intervention in the long term (>1 year) phase [44]. Numerous behaviour change techniques and strategies, such as motivational interviewing and active problem-solving, were recommended, but these generally relied on weaker or less stroke-specific evidence.

### Programme delivery

Eight proximal objectives concerned with programme delivery were identified in the synthesis. These ranged from ensuring that activities are delivered in sufficient quantity to achieve a training effect, to providing programme governance. In total, 57 associated strategies were found. The most commonly recommended and highly evidenced were involving family and carers in the programme, and adapting content and delivery to the individual’s circumstances and goals. However, there was inconsistency between guidelines on a number of recommendations. For instance, group classes conducted in fitness clubs or community centres were recommended by some [35,42], whereas others suggested that individual or home-based training may be preferable, and cited higher level research evidence in support of their recommendation [3,39,48]. Dosage recommendations also differed between guidelines, both in terms of frequency and intensity of exercises. Descriptors for appropriate intensity levels, for example, varied between 'slightly breathless’ [45] and 'aggressive’ [40], the latter level being specifically recommended for longer term stroke survivors [40].

### Synthesis methods

In total, 6 of the 19 included guidelines were found via free text Google searches rather than bibliographic databases. These included the two guidelines providing most of the recommendations on programme delivery [35,42]. Intervention aims were specified in several guidelines and required only minor reformulation to be expressed as target objectives. Objectives were explicitly stated in few guidelines, and most of these referred to the content of programmes rather than their delivery. Therefore, it was necessary to impute several delivery-related objectives. Levels of agreement between the two researchers on the presence or absence of these objectives in four of the guidelines were good, with agreement on all objectives for two of the guidelines [35,40], on 13 out of 14 in one [2] and on 12 out of 14 in another [42].

Explicit links between aims and objectives were rarely made in the guidelines, and none were made in the synthesis. However, strategies were linked to particular content-related objectives in many guidelines, and these linkages were incorporated into the synthesis. Delivery-related objectives were formulated on the basis of themes identified during data extraction and analysis, and so delivery strategies were automatically linked to objectives. Some strategies were thought likely to serve more than one objective but, to avoid duplication, each strategy was assigned to a single objective in the synthesis.

## Discussion

This synthesis, which we refer to as STRIDES (**ST**roke **R**ehabilitation **I**ntervention-**D**evelopment **E**vidence **S**ynthesis), presents an account of the many components that could be included in community-based exercise programmes for stroke survivors. STRIDES represents an exploratory attempt to develop a method of integrating diverse guidelines into a coherent and clinically applicable synthesis. It is novel in a number of respects: selectively abstracting from the guidelines only those recommendations relevant to a particular clinical application; including recommendations that relate not only to the content of the intervention but also to the way it is delivered and to other contextual factors that may significantly influence outcomes; and presenting recommendations in a format particularly suited to programme development. This combination of specificity, inclusivity and integrative restructuring adds value to the published guidelines by providing an account that is more tailored to particular needs and users, and provides a range of solutions to programme delivery issues, which may be selected according to local contexts and requirements. These characteristics can encourage guideline implementation by presenting relevant recommendations in a usable format, and addressing the range of organisational and service delivery issues that programme commissioners and practitioners must deal with.

The synthesis can be used for several purposes. First, to help plan an effective intervention by providing best evidenced suggestions addressing content, delivery issues and other contextual factors. We are currently assessing the value of this synthesis by applying it in the development of an intervention manual for a clinical trial. The synthesis will inform not only the content of the programme but also organisational planning, practitioner training and personalisation of the intervention. By providing guidance on all of these issues and relating them to particular objectives, the synthesis may be useful to others planning similar programmes and so could encourage better uptake of relevant best practice recommendations. Although the STRIDES approach requires further development, its essential components could be applied in the planning of other rehabilitation programmes and other forms of complex intervention. Its feasibility and effectiveness for the purpose requires further evaluation, including resource use compared to “de novo” development of an application-specific guideline.

Second, the synthesis can provide a checklist to evaluate the congruence of existing programmes with best-practice guidelines. We have employed this synthesis to analyse the content of a stroke exercise-based programme manual [50], and the content of exercise programmes informed by the manual (unpublished data). This has allowed us to establish the extent to which this programme meets best-practice guidelines, both in its intended form [50] and its real-world application. The synthesis also enabled identification of potentially novel aspects of the programme. This type of application could be elaborated by developing quality standards, based on the recommendations in the synthesis, to assist formative evaluation of best-practice adherence [51].

The synthesis also enables identification of limitations in guidelines that may affect their implementation. For example, much of cited evidence in this synthesis is derived from studies involving only ambulatory stroke survivors with mild to moderate disabilities, and there is very little reference to those with more severe physical, cognitive and language problems, for whom different strategies may be more feasible or effective. Guideline recommendations that do not take explicit account of the severity of patients’ or clients’ impairments may be rejected by practitioners as impossible to implement [52]. Thus, further research is required to identify strategies that are appropriate for those with more severe impairments. More stroke-specific research is also needed to support recommended strategies concerned with exercise psychology and behaviour change. This is particularly pertinent to those focusing on motivation and self-management skills, both of which may be key to the target outcome of increasing life-long activity levels [53,54]. The inconsistencies between guidelines that were found in some areas may reflect differences in expert opinion where the evidence is scant or of low quality. Robust evidence in these areas may be difficult to obtain [35] and is likely to be context-dependent, but the synthesis includes alternative options suggested in guidelines, and users may choose those they consider most appropriate to their context.

The Intervention Mapping concept provided a useful framework for the synthesis because of its focus on generating an organised programme plan comprising descriptions of target outcomes, proximal objectives and strategies. Since the guidelines differed in scope and language, the imposition of a common terminology was essential. It was necessary to infer proximal objectives for some strategies where these were not explicitly stated. Some degree of conceptual innovation is inevitable in qualitative synthesis [55], and we sought to validate this process by involving several reviewers in the development of themes and terminology, and in the classification of strategies under particular objectives. Other conceptual structures could be used to inform the synthesis, for example the World Health Organisation’s International Classification of Functioning and Disability (ICF) [56]. This employs a biopsychosocial perspective to generate a structured and comprehensive description of the many components of health-state, classified under the domains of body function and structure, activities and participation, and personal and environmental factors. Thus, it can be used to help describe and develop complex health interventions [57]. The ICF emphasises the importance of context and identifies many that relate to service provision, social support and other environmental factors. One of the guidelines included in STRIDES employed the ICF to structure its recommendations [3], and it informed our own thinking during the synthesis process. Sub-sets of ICF classifications have been developed specifically for stroke [58,59] and other health conditions (*e*.*g*., [60,61]), and some studies have suggested personal contextual factors that are not currently classified by the ICF [59,62,63]. This synthesis could be further developed by using the ICF to assess the scope of included guidelines, and to identify areas where new recommendations could be developed.

The Intervention Mapping approach recommends that intervention descriptions specify theoretical mechanisms by which strategies achieve objectives and target outcomes [27]. These were provided in some of the guidelines. Our synthesis could be enhanced by including theoretical mechanisms, as these could assist strategy selection when programmes are being developed with more limited target outcomes, for instance focusing on post-stroke mobility or falls prevention. Information about proposed mechanisms of action might be particularly important in such circumstances, where there may be synergistic relationships between objectives, and strategies may serve multiple objectives.

A number of other issues arose during the synthesis process with implications for its future development and use. Several of the included guidelines were found through free text internet searches, rather than bibliographic or guideline databases. This underlines the value of a broad search strategy, including the use of alternative search terms and databases [64]. Our search may have missed some relevant publications that, along with more recent editions of existing guidelines, could alter the synthesis recommendations. Two guidelines were not accessed because they were not freely available, but we reviewed summaries of these on http://www.guidelines.gov and found no recommendations additional to those in the synthesis. At least one of the included guidelines has recently been updated [4]; however, inspection of the updated version revealed further support for strategies included in the synthesis, rather than suggesting additional ones.

We did not formally rate the quality of included guidelines, though this was variable. Several provided limited or no descriptions of how the evidence they cited had been selected [35,40,42], and so could have been subject to bias. Also, some recommendations were supported by trials of interventions that involved multiple strategies where the effectiveness of a particular component, such as the use of cool-down activities, is unknown [35]. Particularly where there are discrepancies or doubts about particular recommendations, some assessment of the methodological quality of guidelines may help users prioritise recommendations for implementation. Instrument such as the Appraisal of Guidelines, REsearch and Evaluation (AGREE) framework are available to facilitate quality assessment [65]. A review of stroke practice guidelines [66] using the AGREE framework with versions of six of the guidelines included in our synthesis concluded that four of them [2,45,47,48] were of good quality. However, high quality evidence does not necessarily imply relevance or applicability to a particular application or population, and guidelines using lower methodological quality evidence may contain valuable relevant recommendations. Consequently, we would argue that quality appraisal should not be the only criterion used to judge eligibility for inclusion in the synthesis.

## Conclusions

The implementation of practice guidelines may be enhanced by generating accounts that are specific to a particular application and which address not only the content of interventions but also the way they should be delivered, along with other contextual factors that may influence their effectiveness. STRIDES does this by selecting and synthesising relevant best practice recommendations from a range of guidelines, and providing a structured account that can assist those responsible for the development of new programmes and evaluation of existing ones. By including both well-evidenced recommendations and those based on expert consensus, this approach creates a comprehensive description that covers all aspects of programme design. The synthesis also highlights where there is legitimate scope for variation in practice. The methods we have employed require further development, but could be applied to other complex interventions, not only for stroke but for a variety of health conditions.

## Competing interests

CK is a member of the RCP Intercollegiate Stroke Working Party responsible for development of their 2012 stroke guidelines.

An abstract of the early stages of this study has been published [67].

## Authors’ contributions

LP was lead author and reviewer; CA advised on Intervention Mapping and the conceptualisation of the review, and contributed to drafts of paper; AF contributed to drafts of the paper; VG and CK were involved in the review synthesis and contributed to drafts of the paper; RT helped conceptualise the review and contributed to drafts of the paper; SD was involved in conceptualising and conducting the synthesis, and contributed to drafts of the paper. All authors read and approved the final manuscript.

## Supplementary Material

Additional file 1 Guideline search strategies. Click here for file

Additional file 2 Programme proximal objectives and strategies, with guideline page/section references. Click here for file

Additional file 3 Proximal objectives, strategies and evidence levels cited in guidelines. Click here for file
