# Supplementary material for: Synthesising practice guidelines for the development of community-based exercise programmes after stroke
Source: Implement Sci. 2013 Oct 1;8:115. doi: 10.1186/1748-5908-8-115 (PMC3851241; doi:10.1186/1748-5908-8-115)
Supplement: Additional file 1 — Guideline search strategies. [file 1748-5908-8-115-S1.pdf]

Additional File 1

Guideline search strategies

Medline & Cinahl

- ("stroke"[MeSH Terms] OR "stroke"[All Fields])
- AND ("exercise"[MeSH Terms] OR " exercise "[All Fields] OR “rehabilitation”[MeSH Terms] OR " rehabilitation "[All Fields])
- AND "Practice Guideline"[Publication Type]

limits

- 2000 – June 2012
- English language

Guidelines.gov

Keyword: stroke
Guideline Category: Rehabilitation
Publication Year: 2000-2012

Google Scholar

All words in title: practice guideline/s, stroke
Publication year: 2000-2012
