# Supplementary material for: Synthesising practice guidelines for the development of community-based exercise programmes after stroke
Source: Implement Sci. 2013 Oct 1;8:115. doi: 10.1186/1748-5908-8-115 (PMC3851241; doi:10.1186/1748-5908-8-115)
Supplement: Additional file 2 — Programme proximal objectives and strategies, with guideline page/section references. [file 1748-5908-8-115-S2.pdf]

Additional File 2: Programme proximal objectives and strategies, with guideline page/section references

		[1]	[2]	[3]	[4]	[5]	[6]	[7]	[8]	[9]	[10]	[11]	[12]	[13]	[14]	[15]	[16]	[17]	[18]	[19]
	**Objective & Strategy**	Au	Ca1	Ca2	Ca3	Ca4	Ca5	Eu	Ne	NZ	SA	Si	UK1	UK2	UK3	US1	US2	US3	US4	US5
**1**	**Increase muscle strength**	6.2.2		T,p17		P20			E6.4	6.2.2			6.19C		3.6.2.3	p121	T1, p2036		13.1.5	
1a	Lower limb strengthening			T,p17		p11	9.5.3		E6.4						T2,p47		T1, p2036	p2412	13.1.5	
1b	Upper limb strengthening		5.4.1	T,p17		p11	10.2.4		E6.4				6.5.1C		T2,p47		T1, p2036	p2412	13.6	
1c	Trunk and core muscle strengthening			T,p17											T2,p47		T1, p2036			
**2**	**Increase aerobic endurance**		5.4.3	T,p17		p20	9.6; 9.10.16	p103	E6.4	7.8			6.4.1A		3.6	p121	T1, p2036		13.7	
2a	Treadmill training		5.5.3	T,p17		p34			E8.4								T1, p2036		13.5	
2b	Use of static bicycle			T,p17											T2,p47		T1, p2036			
2c	Other large muscle group aerobic activity			T,p17											T2,p47					
**3**	**Regain and maintain normal joint range of movement**			T,p18									6.14.1B		3.6		T1, p2036		13.2	
3a	Lower limb stretching		5.5.2	T,p18											T2,p47					
3b	Upper limb stretching		5.4.1	T,p18			10.11.19		F3.3											
3c	Trunk stretching			T,p18											T2p48					
3d	Lower limb movement through range		5.5.2												T2,p47					
3e	Upper limb movement through range		5.4.3.1		p50										T2,p47					
3f	Unspecified stretch/range of movement exercises			T,p18	p40								6.14.1B; 6.20				T1, p2036			
**4**	**Enhance sensorimotor functions required for functional activity**		5.4.1	T,p18	p50								6.19.1		3.6		T1, p2036			
4a	Standing balance training			T,p18		p45	9.3.1								3.6.2.4				13.4	

4b	Sitting balance training			T,p18		p45			E2.4										13.4	
4c	Aquatic balance training						9.3.1												13.4	
4d	Unspecified balance training						9.10.5						6.18.1A				T1, p2036			
4e	Cyclic movements of paretic arm								F7.4						T2,p47				13.6 do not use	
4f	Proprioceptive & kinaesthetic training		5.4.1		p26	p48			F4.4	6.2.3									14.1	
4g	Mental rehearsal of upper limb movements	6.3.5	5.4.1		p26,43	p14,86	10.2.8; 10.11.9			6.3.4			6.9.1						13.6.5	
**5**	**Enhance functional ability**	1.4.2; 6.3;6.4	5.4.1; 5.5.1	p13	p30	p21			C4.1; E6.4	6.4			6.16		3.6		T1, p2036	p2411	13.1	
5a	Standing up & sitting down practice		5.5.1	T,p17		p45			E4.4	6.3.2			6.16		3.6.2.2					
5b	Step training / stair climbing			T,p17		p45									3.6.2.2		T1, p2036		T15-1, p106	
5c	Walking (including treadmill training)	6.3.4	5.5.3	T,p17		p41	9.10.7; 9.10.8			6.3.2			6.16		3.6.2.2		T1, p2036	2411	13.5	
5d	Walking with rhythmic cueing	6.3.4				p41	9.4; 9.10.11		E14.4	6.3.2									13.5	
5e	Paretic upper limb task-related training	6.3.5	5.4.1		p30, 43,50	p21	10.2.5; 10.11.5		C4.1	6.3.4			6.5.1		3.6.2.3			p2411	13.6	
5f	Bilateral upper limb task-related training	6.3.5				p21				6.3.4			6.5.1						13.6.6	
**6**	**Establish & maintain motivation for regular physical exercise**	1.4.2		p11						1.4.2; 7.8			3.11.1; 6.13.1	12.1	3.5.4					
6a	Address personal beliefs & attitudes														3.2.4					
6b	Promote personal goal-setting									1.7			6.13.1		3.5.2					
6c	Use motivational interviewing												6.13.1							
6d	Promote use of personal reflective diaries														3.5.4					
6e	Promote use of personal exercise record including repetitions, load and time spent														3.5.4					
6f	Use positive feedback												6.13.1							
6g	Emphasise enjoyment			p12											3.3.6					

**7**	**Develop self-management skills for ongoing physical exercise**			p20																
7a	Educate for self-monitoring for adverse events														Box8, p29					
7b	Educate about stroke and stroke recovery patterns																			
7c	Promote active problem-solving									1.7			6.13.1						7.8	
7d	Develop self-efficacy skills			p20									6.13.1							
7e	Encourage self-monitoring to set appropriate exercise levels			p10											3.5.4					
7f	Encourage independent practice of exercises			p20											3.3.2					
7g	Provide guidance booklets														3.3.2					
**8**	**Personalise programme to individual**														3.5.2	p121	T2, p2039	p2433	13.6; 15	
8a	Multidimensional pre-assessment conducted by healthcare professional addressing health status, cautions, contraindications and risks			T1, p5;p7						1.2.2					3.2.2; 3.2.3		T2, p2039	p2410	1.4	
8b	Pre-programme assessment by trainers to enable individualisation of programme				p58										3.5.1					
8c	Adapt programme content to personal situation and goals	6.4		p13; p20						1.7			3.11.1		3.5.2	p121		p2406	4.3;7.5; 15	
8d	Evaluate programme effects on individual, including satisfaction, functional gains, personal goals, resource use, energy levels														3.7.3				7.5; App B, p121	
8e	Supplement group classes with individual sessions														3.3.2					
8f	Sub-divide group classes according to disability levels														3.3.5					
8g	In group classes, conduct functional strengthening exercises together to allow individual monitoring														T2 p47					
8h	Intensity should be adjusted to the individual			p13									5.3.1B		3.6.4.2		T2, p2039			

8i	pre-programme ECG assessment for exercise level																p2035			
8j	If maximal heart-rate is unknown, use low intensity but increase training frequency/duration			p6													T2, p2039			
8k	Shorter, more frequent exercise for frail or deconditioned			p13																
8l	Use of memory aids where necessary									6.6.3			6.30.1		3.5.4					
**9**	**Provide dosage sufficient to establish and maintain benefits**							P102		6.1.1										
9a	at least 3 days/week physical exercise														3.5.3					
9b	20-30 minutes daily moderate intensity physical exercise											p255	5.3.1B							
9c	Progression: increase load / required effort over time	6.2.2		p13									5.3.1B		3.6.2.1				13.1	
9d	Aerobic exercise 20-60 minutes, 3-7 days/week; continuous or accumulated		5.5.3; 5.6														T1, p2036			
9e	Cardiovascular endurance should be large proportion of activity																T1, p2036			
9f	Strengthening exercises: 4-10 types, 2-3 days/week			T,p17											3.6.2.3		T1, p2036			
9g	Flexibility exercises: 2-3 days/week			T,p17													T1, p2036			
9h	Coordination & balance exercises: 2-3 days/week			T,p17													T1, p2036			
9i	Upper limb exercises 1 hour, 6 days/week		5.4.1																	
9j	Warm-up: 15-20 minutes including range of movement and large muscle group activity														3.6.1					
9k	Aerobic warm-up and cool-down, 3-5 minutes at lower intensity			T,p16											3.6.2.2					
9l	Aerobic: up to 10 exercises alternating cardiovascular & local muscle endurance														3.6.2.1					
9m	Include home exercises to increase			p20	p40					6.1.1					3.3.2					

	dose																			
**10**	**Provide context facilitating ongoing regular physical exercise**	1.4.2		p11-12											3.3.4				13.7	
10a	Pre-programme contact to discuss any programme barriers			p12											3.2.1.2		p2036; T2, p2039			
10b	Peer/volunteer to accompany to first one or two sessions														4.2					
10c	Minimal use of equipment to facilitate home practice														3.6.2.1					
10d	Promote family / carer involvement	6.1.1		T1,p9	p40					6.1.1; 8.8	13.3		3.11.1; 7.5.1			p114	p2036; T2, p2039	p2431	7.6;17	
10e	Use peer mentoring									8.7					3.3.6					
10f	Use group format for social support														3.3.2					
10g	Provide opportunities to socialise before and after training														3.5.5					
10h	Use of mixed media including internet-based and tele-training									1.2.5									8.1	2644
10i	Locate at home or centre according to personal circumstances / preferences	1.4.1			p40		7.2.4;7.3.7									p121			7.8; 13.7	
10j	Locate in own residential environment					p21			C4.2		p778									
10k	Provision of transport where necessary, or locate near good public transport links	1.4.2		p12									7.2.1		3.1.5					
10l	Convenient time			p12																
10m	Ongoing programme provision			p12											3.3.4					
10n	Sign-post to other relevant services / facilities	8.6.1								8.7			7.3.1						7.8	
**11**	**Ensure adequate staffing numbers to provide safe and effective training**							P101							3.3.3					
11a	Instructor: participant ratio: 1:3 to 1:5			p11																
11b	Instructor: participant ratio: up to 1:8 depending on mix & time since														3.3.3					

	started exercising																			
11c	Supernumerary volunteers or trainees to take part in sessions														4.5					
**12**	**Ensure staff are adequately trained for client group**														3.4					
12a	Delivered by instructors with knowledge and training in exercise and stroke			p21										12.9	3.2.5.3					
12b	Provide in-service training to instructors														3.2.5.3					
12c	Ensure stroke-awareness training of frontline staff in course venue														4.3					
**13**	**Integrate programme into stroke pathway**					No evid p84									3.2; 3.3			p2404		
13a	Develop partnership agreements between stakeholders														3.1.3					
13b	Referral by healthcare practitioner using clear eligibility criteria			p5											3.2.1.1 3.2.2.3 3.2.3					
13c	Encourage referring practitioner to visit programme														3.3.1					
13d	Established procedures for transferring responsibilities from referrers to trainers														3.2.5					
13e	Ongoing communication with (and feedback to) other stakeholders including health care professionals, service commissioners, local stroke networks														3.7.5					
13f	Referral for other treatments where appropriate														3.3.7; 4.4	p121				
**14**	**Ensure adequate programme governance**														3.1					
14a	Overseen by management group														3.1.1					
14b	Plan for programme evaluation			p20											3.7					
14c	Have procedures for recording and reporting adverse events			p20											3.7					
14d	Have data protection procedures														3.7					

14e	Obtain and check ongoing consent			p5,11											3.2.5.4<br>3.2.3.3					

References

1. National Stroke Foundation: **Clinical Guidelines for Stroke 2010**. Melbourne: National Stroke Foundation; 2010.
2. Canadian Stroke Network: **Canadian Best Practice Recommendations for Stroke Care**. Ottawa: Canadian Stroke Network; 2010.
3. Ontario Stroke Network: **Guidelines for Community Based Exercise Programs for People with Stroke**. Toronto: Ontario Stroke Network; 2010.
4. Consensus Panel on the Management of the Hemiplegic Arm and Hand: **Management of the Post Stroke Arm and Hand: Treatment Recommendations of the 2001 Consensus Panel**. Hamilton, Ontario: Heart and Stroke Foundation of Ontario; 2001.
5. Ottawa Panel: **Ottawa panel evidence-based clinical practice guidelines for post-stroke rehabilitation**. *Top Stroke Rehabil* 2006, **13**(2):1-269.
6. **Evidence-Based Review of Stroke Rehabilitation** [www.ebrsr.com/]
7. Quinn TJ, Paolucci S, Sunnerhagen KS, Sivenius J, Walker MF, Toni D, Lees KR: **Evidence-based stroke rehabilitation: an expanded guidance document from the european stroke organisation (ESO) guidelines for management of ischaemic stroke and transient ischaemic attack 2008**. *J Rehabil Med* 2009, **41**(2):99-111.
8. van Peppen RPS, Kwakkel G, Harmeling-van der Wel BC, Kollen BJ, Hobbelen JSM, Buurke JH: **KNGF Clinical Practice Guideline for physical therapy in patients with stroke. Review of the evidence. [Translation 2008].** *Nederlands Tijdschrift voor Fysiotherapie* 2004, **114**(5):(suppl).
9. Stroke Foundation of New Zealand and New Zealand Guidelines Group: **Clinical Guidelines for Stroke Management 2010**. Wellington: Stroke Foundation of New Zealand; 2010.
10. Bryer A, Connor M, Haug P, Cheyip B, Staub H, Tipping B, Duim W, Pinkney-Atkinson V: **South African guideline for management of ischaemic stroke and transient ischaemic attack 2010: a guideline from the South African Stroke Society (SASS) and the SASS Writing Committee**. *S Afr Med J* 2010, **100**(11 Pt 2):747-778.
11. Venketasubramanian N, Pwee KH, Chen CP: **Singapore ministry of health clinical practice guidelines on stroke and transient ischemic attacks**. *Int J Stroke* 2011, **6**(3):251-258.
12. RCP: **National Clinical Guideline for Stroke**. 3rd edition. London: Royal College of Physicians; 2008.
13. SIGN: **Management of patients with stroke or TIA: assessment, investigation, immediate management and secondary prevention. A national clinical guideline**. Edinburgh: Scottish Intercollegiate Guidelines Network; 2008.
14. Best C, van Wijck F, Dinan-Young S, Dennis J, Smith M, Frazer H, Donaghy M, Meda G: **Best Practice Guidance for the Development of Exercise after Stroke Services in Community Settings**. University of Edinburgh; 2010.
15. Duncan PW, Zorowitz R, Bates B, Choi JY, Glasberg JJ, Graham GD, Katz RC, Lamberty K, Reker D: **Management of Adult Stroke Rehabilitation Care: a clinical practice guideline**. *Stroke* 2005, **36**(9):e100-143.

16. Gordon NF, Gulanick M, Costa F, Fletcher G, Franklin BA, Roth EJ, Shephard T: **Physical activity and exercise recommendations for stroke survivors: an American Heart Association scientific statement**. *Stroke* 2004, **35**(5):1230-1240.
17. Miller EL, Murray L, Richards L, Zorowitz RD, Bakas T, Clark P, Billinger SA: **Comprehensive overview of nursing and interdisciplinary rehabilitation care of the stroke patient: a scientific statement from the American Heart Association**. *Stroke* 2010, **41**(10):2402-2448.
18. Department of Veteran Affairs: **VA/DOD Clinical practice guideline for the management of stroke rehabilitation**. *J Rehabil Res Dev* 2010, **47**(9):1-43.
19. Schwamm LH, Audebert HJ, Amarenco P, Chumbler NR, Frankel MR, George MG, Gorelick PB, Horton KB, Kaste M, Lackland DT *et al*: **Recommendations for the implementation of telemedicine within stroke systems of care: a policy statement from the American Heart Association**. *Stroke* 2009, **40**(7):2635-2660.
