# Supplementary material for: Synthesising practice guidelines for the development of community-based exercise programmes after stroke
Source: Implement Sci. 2013 Oct 1;8:115. doi: 10.1186/1748-5908-8-115 (PMC3851241; doi:10.1186/1748-5908-8-115)
Supplement: Additional file 3 — Proximal objectives, strategies and evidence levels cited in guidelines. [file 1748-5908-8-115-S3.pdf]

<u>Additional File 3: Proximal objectives, strategies and evidence levels cited in guidelines.</u>

Abbreviations: App – appendix; Cons – expert consensus; GL – guidelines; LT – long term; RCT – randomised control trial; SR – systematic review

Different systems of rating evidence were used in guidelines. See individual guidelines for system employed. Where evidence was not rated by guideline, the type of evidence cited is identified.
Levels of evidence for recommendations in one guideline [7] are provided in a related publication by the same organisation [20]

		Au	Ca1	Ca2	Ca3	Ca4	Ca5	Eu	Ne	NZ	SA	Si	UK1	UK2	UK3	US1	US2	US3	US4	US5
	**Objective & Strategy**	[1]	[2]	[3]	[4]	[5]	[6]	[7] [20]	I[8]	[9]	[10]	[11]	[12]	[13]	[14]	[15]	[16]	[17]	[18]	[19]
**1**	**Increase muscle strength**	SR		other GLs		I			1	B			SRs		SR	II-2	other GLs		I	
1a	Lower limb strengthening			other GLs		I	conflicting		1						part RCT		other GLs	1		
1b	Upper limb strengthening		A (LT)	other GLs			1a		unclear				SR		part RCT		other GLs	1	I	
1c	Trunk and core muscle strengthening			other GLs											RCT		other GLs			
**2**	**Increase aerobic endurance**		B (LT)	A			1a	SR	3	A			SR		SR	II-2	other GLs		I	
2a	Treadmill training		B (LT)	other GLs		I	1a		1								other GLs		I	
2b	Use of static bicycle			other GLs			1a										other GLs			
2c	Other large muscle group aerobic activity			other GLs											part RCT					
**3**	**Regain and maintain normal joint range of movement**		C										cons; SR		part RCT		other GLs		I	
3a	Lower limb stretching		RCT	other GLs											part RCT					
3b	Upper limb stretching		C (LT)	other GLs			1a		2											
3c	Trunk stretching			other GLs											part RCT					
3d	Lower limb movement through range		C												part RCT					
3e	Upper limb movement through range		C (LT)		cons										part RCT					
3f	Unspecified stretch/range of movement exercises			other GLs	cons								cons; SR				other GLs			

**4**	**Enhance sensorimotor functions required for functional activity**		A (LT)		I								cons				other GLs		III	
4a	Standing balance training			other GLs			1a								RCT (other condition)				I	
4b	Sitting balance training			other GLs					1										I	
4c	Aquatic balance training						1b												I	
4d	Unspecified balance training												SRs				other GLs			
4e	Cyclic movements of paretic arm								3						part RCT					
4f	Proprioceptive & kinaesthetic training		B (LT)		cons	II			3	C										
4g	Mental rehearsal of upper limb movements	SR	B (LT)		cons	multiple RCTs	conflicting			B			SR						I	
**5**	**Enhance functional ability**	B	A (LT)		cons				1	B							other GLs	1	I	
5a	Standing up & sitting down practice		RCTs	other GLs					1	A			SR		part RCT					
5b	Step training / stair climbing			other GLs											part RCT		other GLs			
5c	Walking (including treadmill training)	SR	B (LT)	other GLs			1a		1	A			SR		SRs		other GLs	1	I	
5d	Walking with rhythmic cueing	SR					1a		2	B									I	
5e	Paretic upper limb task-related training	SR	A (LT)		II		1a		1	C			SR		part RCT			1		
5f	Bilateral upper limb task-related training	SR					conflicting			C			SR					1	I	
**6**	**Establish & maintain motivation for regular physical exercise**	C		cons						C			cons	2	cons					
6a	Address personal beliefs & attitudes														RCT: advice insufficient					
6b	Promote personal goal-setting									cons			SR not stroke specific		cons					
6c	Use motivational interviewing												Part of RCT							
6d	Promote use of personal reflective														cons					

	diaries																			
6e	Promote use of personal exercise record including repetitions, load and time spent														cons					
6f	Use positive feedback												Part of RCT							
6g	Emphasise enjoyment			cons											cons					
**7**	**Develop self-management skills for ongoing physical exercise**									cons										
7a	Educate for self-monitoring for adverse events														App1					
7b	Educate about stroke and stroke recovery patterns																			
7c	Promote active problem-solving									cons			cons							
7d	Develop self-efficacy skills												cons; part of RCT							
7e	Encourage self-monitoring to set appropriate exercise levels														cons					
7f	Encourage independent practice of exercises														cons					
7g	Provide guidance booklets														cons					
**8**	**Personalise programme to individual**														cons	cons	cons		I	
8a	Multidimensional pre-assessment conducted by healthcare professional addressing health status, cautions, contraindications and risks			cons						cons					cons		cons	IIA	III	
8b	Pre-programme assessment by trainers to enable individualisation of programme				cons										cons					
8c	Adapt programme content to personal situation and goals	B		other GLs						C					cons	cons				
8d	Evaluate programme effects on individual, including satisfaction, functional gains, personal goals, resource use, energy levels														cons				cons	
8e	Supplement group classes with individual sessions														cons					
8f	Sub-divide group classes according to disability levels														cons					
8g	In group classes, conduct functional strengthening exercises together to														cons					

	allow individual monitoring																			
8h	Intensity should be adjusted to the individual			other GLs									cons		cons		cons			
8i	pre-programme ECG assessment for exercise level																cons			
8j	If maximal heart-rate is unknown, use low intensity but increase training frequency/duration			other GLs													cons			
8k	Shorter, more frequent exercise for frail or deconditioned			other GLs																
8l	Use of memory aids where necessary									D			cons		cons					
**9**	**Provide dosage sufficient to establish and maintain benefits**							II		cons										
9a	at least 3 days/week physical exercise														SR					
9b	20-30 minutes daily moderate intensity physical exercise											cons	cons		cons					
9c	Progression: increase load / required effort over time	SRs		other GLs									cons		cons				I	
9d	Aerobic exercise 20-60 minutes, 3-7 days/week; continuous or accumulated		B														other GLs			
9e	Cardiovascular endurance should be large proportion of activity																other GLs			
9f	Strengthening exercises: 4-10 types, 2-3 days/week			other GLs											cons		other GLs			
9g	Flexibility exercises: 2-3 days/week			other GLs													other GLs			
9h	Coordination & balance exercises: 2-3 days/week			other GLs													other GLs			
9i	Upper limb exercises 1 hour, 6 days/week		C (LT)																	
9j	Warm-up: 15-20 minutes including range of movement and large muscle group activity														part RCT					
9k	Aerobic warm-up and cool-down, 3-5 minutes at lower intensity			other GLs											part RCT					
9l	Aerobic: up to 10 exercises alternating cardiovascular & local muscle endurance														cons					
9m	Include home exercises to increase dose			other GLs	cons					cons					cons					
**10**	**Provide context facilitating ongoing**	RCT													cons				SR-A	

	**regular physical exercise**																			
10a	Pre-programme contact to discuss any programme barriers			other GLs											cons		cons			
10b	Peer/volunteer to accompany to first one or two sessions														cons					
10c	Minimal use of equipment to facilitate home practice														cons					
10d	Promote family / carer involvement	cons		other GLs	cons					cons	IV		RCTs			III	other GLs	I-IIa	I	
10e	Use peer mentoring									cons					RCT (other conditions)					
10f	Use group format for social support														Qualit-ative					
10g	Provide opportunities to socialise before and after training														cons					
10h	Use of mixed media including internet-based and tele-training									C										IIa
10i	Locate at home or centre according to personal circumstances / preferences	B			cons		1a									II-2				
10j	Locate in own residential environment					1 RCT home based			1		IV									
10k	Provision of transport where necessary, or locate near good public transport links	cons											cons		Qualit-ative study					
10l	Convenient time																			
10m	Ongoing programme provision														RCTs (benefits don't last)					
10n	Sign-post to other relevant services / facilities	cons								cons			cons							
**11**	**Ensure adequate staffing numbers to provide safe and effective training**							II							cons					
11a	Instructor: participant ratio: 1:3 to 1:5			Seen in RCTs																
11b	Instructor: participant ratio: up to 1:8 depending on mix & time since started exercising														cons					

11c	Supernumerary volunteers or trainees to take part in sessions														cons					
**12**	**Ensure staff are adequately trained for client group**														cons					
12a	Delivered by instructors with knowledge and training in exercise and stroke			cons										cons	cons					
12b	Provide in-service training to instructors														cons					
12c	Ensure stroke-awareness training of frontline staff in course venue														cons					
**13**	**Integrate programme into stroke pathway**					No evidence p84									cons					
13a	Develop partnership agreements between stakeholders														cons					
13b	Referral by healthcare practitioner using clear eligibility criteria			Other GLs											cons					
13c	Encourage referring practitioner to visit programme														cons					
13d	Established procedures for transferring responsibilities from referrers to trainers														cons					
13e	Ongoing communication with (and feedback to) other stakeholders including health care professionals, service commissioners, local stroke networks														cons					
13f	Referral for other treatments where appropriate														cons	cons				
**14**	**Ensure adequate programme governance**														cons					
14a	Overseen by management group														cons					
14b	Plan for programme evaluation														cons					
14c	Have procedures for recording and reporting adverse events														cons					
14d	Have data protection procedures														cons					
14e	Obtain and check ongoing consent														cons					

<u>**References**</u>

1. National Stroke Foundation: **Clinical Guidelines for Stroke 2010**. Melbourne: National Stroke Foundation; 2010.
2. Canadian Stroke Network: **Canadian Best Practice Recommendations for Stroke Care**. Ottawa: Canadian Stroke Network; 2010.
3. Ontario Stroke Network: **Guidelines for Community Based Exercise Programs for People with Stroke**. Toronto: Ontario Stroke Network; 2010.
4. Consensus Panel on the Management of the Hemiplegic Arm and Hand: **Management of the Post Stroke Arm and Hand: Treatment Recommendations of the 2001 Consensus Panel**. Hamilton, Ontario: Heart and Stroke Foundation of Ontario; 2001.
5. Ottawa Panel: **Ottawa panel evidence-based clinical practice guidelines for post-stroke rehabilitation**. *Top Stroke Rehabil* 2006, **13**(2):1-269.
6. **Evidence-Based Review of Stroke Rehabilitation** [www.ebrsr.com/]
7. Quinn TJ, Paolucci S, Sunnerhagen KS, Sivenius J, Walker MF, Toni D, Lees KR: **Evidence-based stroke rehabilitation: an expanded guidance document from the european stroke organisation (ESO) guidelines for management of ischaemic stroke and transient ischaemic attack 2008**. *J Rehabil Med* 2009, **41**(2):99-111.
8. van Peppen RPS, Kwakkel G, Harmeling-van der Wel BC, Kollen BJ, Hobbelen JSM, Buurke JH: **KNGF Clinical Practice Guideline for physical therapy in patients with stroke. Review of the evidence. [Translation 2008].** *Nederlands Tijdschrift voor Fysiotherapie* 2004, **114**(5):(suppl).
9. Stroke Foundation of New Zealand and New Zealand Guidelines Group: **Clinical Guidelines for Stroke Management 2010**. Wellington: Stroke Foundation of New Zealand; 2010.
10. Bryer A, Connor M, Haug P, Cheyip B, Staub H, Tipping B, Duim W, Pinkney-Atkinson V: **South African guideline for management of ischaemic stroke and transient ischaemic attack 2010: a guideline from the South African Stroke Society (SASS) and the SASS Writing Committee**. *S Afr Med J* 2010, **100**(11 Pt 2):747-778.
11. Venketasubramanian N, Pwee KH, Chen CP: **Singapore ministry of health clinical practice guidelines on stroke and transient ischemic attacks**. *Int J Stroke* 2011, **6**(3):251-258.
12. RCP: **National Clinical Guideline for Stroke**. 3rd edition. London: Royal College of Physicians; 2008.
13. SIGN: **Management of patients with stroke or TIA: assessment, investigation, immediate management and secondary prevention. A national clinical guideline**. Edinburgh: Scottish Intercollegiate Guidelines Network; 2008.
14. Best C, van Wijck F, Dinan-Young S, Dennis J, Smith M, Frazer H, Donaghy M, Meda G: **Best Practice Guidance for the Development of Exercise after Stroke Services in Community Settings**. University of Edinburgh; 2010.
15. Duncan PW, Zorowitz R, Bates B, Choi JY, Glasberg JJ, Graham GD, Katz RC, Lamberty K, Reker D: **Management of Adult Stroke Rehabilitation Care: a clinical practice guideline**. *Stroke* 2005, **36**(9):e100-143.
16. Gordon NF, Gulanick M, Costa F, Fletcher G, Franklin BA, Roth EJ, Shephard T: **Physical activity and exercise recommendations for stroke survivors: an American Heart Association scientific statement**. *Stroke* 2004, **35**(5):1230-1240.
17. Miller EL, Murray L, Richards L, Zorowitz RD, Bakas T, Clark P, Billinger SA: **Comprehensive overview of nursing and interdisciplinary rehabilitation care of the stroke patient: a scientific statement from the American Heart Association**. *Stroke* 2010, **41**(10):2402-2448.
18. Department of Veteran Affairs: **VA/DOD Clinical practice guideline for the management of stroke rehabilitation**. *J Rehabil Res Dev* 2010, **47**(9):1-43.

19. Schwamm LH, Audebert HJ, Amarenco P, Chumbler NR, Frankel MR, George MG, Gorelick PB, Horton KB, Kaste M, Lackland DT *et al*: **Recommendations for the implementation of telemedicine within stroke systems of care: a policy statement from the American Heart Association**. *Stroke* 2009, **40**(7):2635-2660.
20. European Stroke Organisation: **Guidelines for management of ischaemic stroke and transient ischaemic attack 2008**. *Cerebrovasc Dis* 2008, **25**(5):457-507.
